# Ultrathin nickel hydroxide nanosheet arrays grafted biomass-derived honeycomb-like porous carbon with improved electrochemical performance as a supercapacitive material

**DOI:** 10.1038/srep45201

**Published:** 2017-03-24

**Authors:** Goli Nagaraju, Sung Min Cha, Jae Su Yu

**Affiliations:** 1 Department of Electronic Engineering, Institute for Wearable Convergence Electronics, Kyung Hee University, 1 Seocheon-dong, Giheung-gu, Yongin-si, Gyeonggi-do 446-701, Republic of Korea

## Abstract

Three-dimensional hierarchical honeycomb-like activated porous carbon pillared ultrathin Ni(OH)_2_ nanosheets (Ni(OH)_2_ NSs@HAPC) for use as supercapacitor materials were facilely synthesized. With an aid of pine cone flowers as a biomass source, HAPC conducting scaffolds were prepared by the alkali treatment and pyrolysis methods under an inert gas atmosphere. Subsequently, the Ni(OH)_2_ NSs were synthesized evenly on the surface of HAPC via a solvothermal method. The resulting HAPC and Ni(OH)_2_ NSs@HAPC composite materials offered free pathways for effective diffusion of electrolyte ions and fast transportation of electrons when employed as an electrode material. The Ni(OH)_2_ NSs@HAPC composite electrode exhibited excellent electrochemical properties including a relatively high specific capacitance (C_sp_) value of ~ 916.4 F/g at 1 A/g with good cycling stability compared to the pristine HAPC and Ni(OH)_2_ NSs electrodes. Such bio-friendly derived carbon-based materials with transition metal hydroxide/oxide composite materials could be a promising approach for high-performance energy storage devices because of their advantageous properties of cost effectiveness and easy availability.

In the face of increasing energy concerns and global warming issues resulting from the burning of fossil fuels, environmentally benign and sustainable power sources are enforced to overcome the global needs^1^. Consequently, research interest has been increased day by day to develop renewable and sustainable energy storage systems^2^,^3^,^4^,^5^,^6^. Among the explored energy storage systems, supercapacitors/ultracapacitors which can bridge the critical performance gap between energy density in lithium-ion batteries and high power density in classical dielectric capacitors are considered as the most promising and important energy storage device^7^,^8^,^9^,^10^. Especially, supercapacitors have shown beneficial properties of long working lifetime, easy fabrication, fast recharge ability, low cost and safe operation compared to any other energy storage systems, which makes them suitable for several applications^11^,^12^,^13^. In terms of the energy storage process in supercapacitors, they can be divided into two types; one is electric double layer capacitors (EDLCs) and the other is pseudocapacitors. In EDLCs, electrical energy is stored by the electrostatic accumulation of charges in the electric double layer near the interface of electrode-electrolyte. In contrast, pseudocapacitors follow the pseudocapacitive mechanism, where the fast and reversible Faradaic redox reactions occur on the surface of electroactive materials^14^,^15^.

Many kinds of electrodes and electroactive materials have been widely explored in supercapacitors^16^. Typically, the carbon-based materials (such as activated carbon and graphene materials) in EDLCs and transition metal oxides/hydroxide (Co_3_O_4_, MnO_2_, NiCo_2_O_4_, Co(OH)_2_, Ni(OH)_2_, etc.) in pseudocapacitors are being extensively investigated because of their low cost, less toxicity and versatility in structure and morphology^17^,^18^,^19^,^20^,^21^,^22^,^23^,^24^,^25^. Despite the significant developments achieved in electroactive materials for EDLCs and pseudocapacitors, the suitability of these materials for practical applications is still limited because of their poor specific capacitance in carbon-based materials, while the relatively low power density and poor cycling stability usually occur in transition metal oxide/hydroxides^3^,^14^,^26^. To overcome these problems, recently, synergistic effects of collective EDLCs-type carbon-based materials with transition metal oxide/hydroxide materials have been considered. Since these composite materials are expected to improve electrical conductivity and porous structure, which results in the enhanced electrochemical performance in supercapacitors^27^,^28^,^29^,^30^,^31^. Notably, the composite electroactive materials prepared using biomass-derived activated carbon materials with cost-effective transition metal hydroxide nanostructured materials are highly desirable to meet the urgent need for green and sustainable supercapacitors^32^. However, the studies on the preparation of highly conductive composite materials with biomass-derived activated carbon and transition metal hydroxide nanomaterials are still few.

As a natural lignocellulosic biopolymer, pine cone flowers, i.e., a woody fruit of pine tree (a family of pinaceae), contain mainly glucose, mannose, klason lignin and small amounts of galactose/xylose derivate^33^,^34^. Such abundant carbon derivatives of the pine cone flowers are expected to produce biomass-based activated carbon materials. On the other hand, nickel hydroxide (Ni(OH)_2_) is considered as a promising electroactive material among various transition metal oxide/hydroxides due to simple synthesis process, versatile morphologies and well-defined electrochemical redox activity in aqueous electrolyte solutions^23^,^35^,^36^,^37^. In this work, we employed pine cone flowers as the biomass source to prepare the honeycomb-like activated porous carbon (HAPC) using the alkali treatment and pyrolysis techniques. Simultaneously, the Ni(OH)_2_ nanosheets (NSs) were uniformly covered over the conductive scaffolds of HAPC to enhance the synergistic effect in Ni(OH)_2_ NSs@HAPC composite materials. When utilized as the electroactive material for supercapacitors, the composite materials exhibited relatively higher energy storage properties than the pristine HAPC and Ni(OH)_2_ NSs.

## Experimental details

### Chemicals and materials

The hybrid pine cone flowers from pine trees were collected inside the Yeongtong-gu public park, South Korea. Nickel nitrate hexahydrate (Ni(NO_3_)_2_·6H_2_O), hexamethylenetetramine (HMTA, C_6_H_12_N_4_) and polyvinylidene fluoride (PVdF, −(C_2_H_2_F_2_)_n_−) were purchased from Sigma-Aldrich Co., South Korea. Potassium hydroxide (KOH), hydrochloric acid (HCl) and N-methyl-2-pyrrolidone (NMP, C_5_H_9_NO) were obtained from Daejung Chemicals Ltd., South Korea. Carbon cloth (CC) is commercially available from Nara Cell-Tech Co., South Korea. All the chemicals were of analytical grade purity and were used as received without any further purification.

### Synthesis of HAPC from pine cone flowers

The HAPC samples were prepared by a facile alkali treatment and carbonization process of pine cone flowers^38^. At first, the pine cone flowers were collected in a glass beaker and thoroughly washed with ethanol and de-ionized (DI) water to remove the adhered impurities and dust particles. Subsequently, the washed pine cone flowers were dried in sunlight and then in an oven at 70 °C overnight. The as-dried pine cone flowers became rigid and were mechanically pulverized into a fine powder by using a mixer (Philips, HR2100). For alkali treatment process, an aqueous solution of KOH was prepared by adding ∼ 5.0 g of KOH flakes into 80 ml of DI water. Then, the equal amount of pine cone powder (0.5 g) was introduced into the KOH solution and heated at the reflux temperature under continuous magnetic stirring for 10 h. Consequently, the dark brown colloidal solution was observed. The residue of the mixture was separated by centrifugation, washed and vacuum-dried at 80 °C overnight. Then, the KOH treated powder was carbonized at 900 °C in the tubular furnace under a constant flow of argon gas for 2 h to obtain the activated carbon sample. Herein, the corresponding heating rate was maintained at 5 °C/min. The resulting carbon samples were neutralized with 1 M of HCl solution and washed several times with DI water to ensure a complete removal of the potassium salt. By the subsequent centrifugation and drying at 70 °C overnight, the pure HAPC powdered samples were collected. Meanwhile, the carbon powder was also prepared using pine cone powder without KOH treatment (named it as PC).

### Fabrication of Ni(OH)_2_ NSs@HAPC composites

The Ni(OH)_2_ NSs coated HAPC composites were synthesized by a facile solvothermal method at low temperature. To synthesize the Ni(OH)_2_ NSs@HAPC composites, initially, 40 mg of the as-prepared HAPC sample was dispersed in 20 ml of ethanol under ultrasonication treatment for 30 min. And, 0.39 g of Ni(NO_3_)_2_·6H_2_O was dissolved in a beaker containing 20 ml of DI water. After that, a transparent green color solution of Ni(NO_3_)_2_·6H_2_O was added dropwise to the HAPC solution under magnetic stirring. Subsequently, appropriate amount of C_6_H_12_N_4_ and a few drops of ammonia solution were also added to the above solution. The resultant mixture was vigorously stirred on hotplate at room temperature for 1 h to achieve a homogeneous solution. Then, the growth solution was transferred into a Teflon-lined stainless steel autoclave system and heated to 90 °C for 10 h. After that, the autoclave was left to cool down to room temperature. As a result, a greenish-black color precipitate from autoclave was obtained, which was filtered and washed with DI water several times to remove the impurities. Finally, the powdered sample of the Ni(OH)_2_ NSs@HAPC composites was obtained by drying at 70 °C for 6 h. For comparison, the pristine Ni(OH)_2_ was also prepared using the same procedure without adding the HAPC powder.

### Characterizations

The structure and morphology of the prepared samples were investigated by using a field-emission scanning electron microscope (FE-SEM, FE-SEM; Carl Zeiss, LEO SUPRA 55) equipped with energy-dispersive X-ray spectroscopy (EDX) and a transmission electron microscope (TEM; JEM 200CX, JEOL). The crystallographic nature of the materials was analyzed by X-ray diffraction (XRD, M18XHF-SRA, MacScience Ltd.). The presence of carbon in the prepared material was studied by Raman spectroscopy (HR-Raman Spectrometer, inVia). X-ray photoelectron spectroscopy (XPS, Thermo Electron MultiLab2000) with Al Kα radiation was carried out to investigate the surface functional groups of the samples. The surface area and porosity of the sample were evaluated by BET analysis (BELSORP-max (MP)).

### Electrochemical measurements

Prior to the electrode fabrication, the CC was treated in 1 M of HNO_3_ solution at 80 °C for 3 h, gently rinsed with DI water and dried under a flow of nitrogen (N_2_) gas. To coat the active materials on the CC, first, 80% of the prepared HAPC sample was placed in an agate mortar and finely grounded into a smooth powder. Then, 10% of PVdF, 10% of super P carbon black and required amount of NMP solvent were added into the HAPC powder. The resultant suspension was thoroughly mixed with agate mortar to form a homogeneous slurry. Thereafter, the slurry was uniformly coated onto the CC electrode, and it was air-dried for 1 h and further oven-dried at 60 °C for 3 h to completely evaporate the solvent. Similarly, Ni(OH)_2_ NSs@HAPC and Ni(OH)_2_ NSs active materials were also coated on the CC by the above procedure. The mass of the active materials was determined by using a high-resolution microbalance (OHAUS DV214C). For electrochemical measurements, three electrodes in a glass beaker cell were used with an active materials coated CC as the working electrode, a Pt wire counter electrode and a KCL saturated Ag/AgCl reference electrode, respectively. The electrolyte was composed of 1 M of aqueous KOH solution. Cyclic voltammetry (CV), galvanic charge-discharge (GCD) measurements and electrochemical impedance spectroscopy (EIS) were performed by using an IVIUMSTAT (The Netherlands) electrochemical interface system. The specific capacitance of the electrode materials was measured by GCD curves and can be estimated based on the following equation:^25^


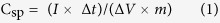


Here, C_sp_ (F/g) is the specific capacitance, I (A) is the discharge current, ∆t (s) is the discharge time, ∆V (V) is the potential range and m (g) is the mass loading of the electroactive materials on CC.

## Results and Discussion

**Figure 1:**
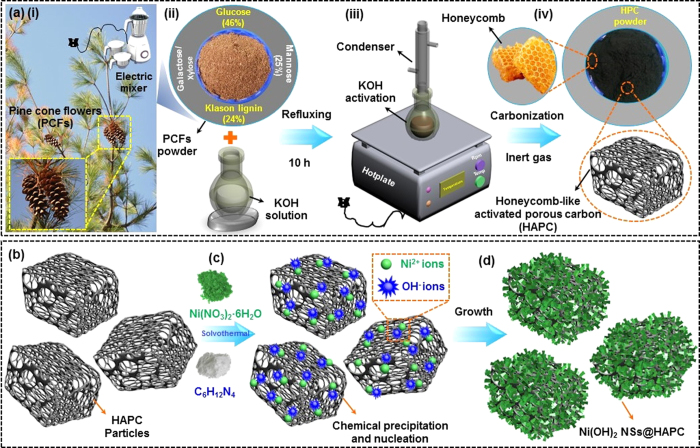
Schematic illustration of the synthesis process of (**a**) HAPC and (**b**–**d**) Ni(OH)_2_ NSs@HAPC composite materials using pyrolysis and solvothermal methods.

Figure 1 illustrates the schematic diagram for the preparation process of the pine cone flowers derived HAPC and the Ni(OH)_2_ NSs@HAPC composites. As a typical lignocellulosic biomass source, the pine cone flowers were mainly composed of natural biopolymers, such as glucose (46%), mannose (25%), klason lignin (24%) and small amounts of galactose/xylose, as shown in Fig. 1(a)(i) and (a)(ii). Such abundant cellulosic and lignin derivatives in pine cone flowers could serve as an excellent biomass source for preparing the activated carbon samples. For attaining the large surface area and porous property in carbon-based materials, the alkali treatment is one of the important processes. Accordingly, the pulverized powder of pine cone flowers was first chemically treated with KOH solution (Fig. 1(a)(iii)). Due to the porous nature, the KOH species easily permeated into the finely grounded pine cone powder. After the chemical treatment, the residue of the chemically activated material was separated by centrifugation and dried at ambient temperature, respectively. The dried powder was then carbonized at 900 °C to obtain hierarchical and honeycomb structured activated carbon samples (i.e., HAPC) as depicted in Fig. 1(a)(iii). Typically, the KOH plays an important role in forming the highly porous and hollow structured materials from the biomass source. The as-obtained HAPC acts as an ideal scaffold for the growth of ultrathin Ni(OH)_2_ NSs by a simple solvothermal method (Fig. 1(b–d)). Herein, the growth of Ni(OH)_2_ NSs on HAPC involves the nucleation, chemical precipitation and crystallization processes. The reaction during the synthesis process is described as follows:^39^


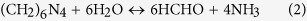



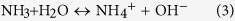



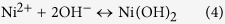


In general, HMTA acts as a hydrolyzing agent and pH buffer, which initiates the slow release of hydroxyl (OH^−^) ions through formaldehyde (HCHO) and ammonia (NH_3_) decomposition, respectively (Equations (2) and (3)). Such slowly released OH^−^ ions play a crucial role in the formation of Ni(OH)_2_ NSs on HAPC (i.e., Ni(OH)_2_ NSs@HAPC) by the homogeneous nucleation and crystallization processes with nickel (Ni^2+^) ions in the growth solution (Equation (4)).

**Figure 2:**
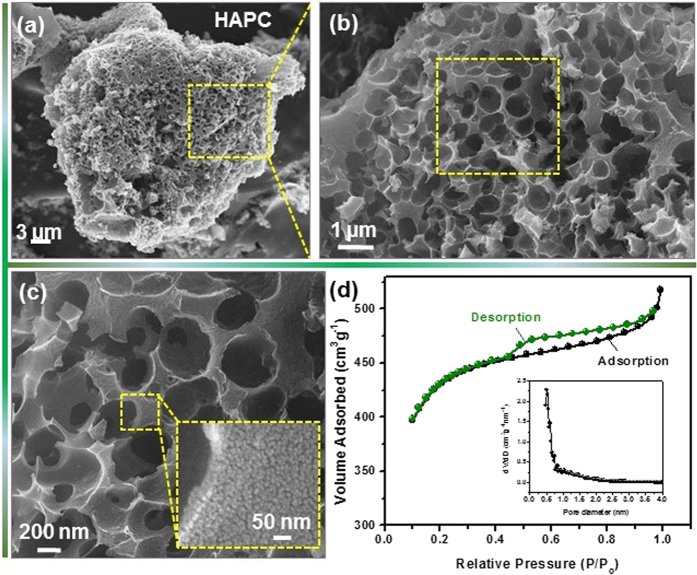
(**a**–**c**) FE-SEM images and (**b**) BET results of the HAPC obtained after chemical activation and carbonization processes of pine cone flowers.

Figure 2 shows the morphology and structure of the as-prepared HAPC. After alkali treatment and pyrolysis processes, the prepared powder exhibited the natural honeycomb-like structure as shown in the low-magnification FE-SEM image of Fig. 2(a) and TEM image of Fig. S1(a). With the increased magnification in Fig. 2(b), HAPC showed a three-dimensional (3D) interconnected structure with macropore sizes of ∼ 400–600 nm. In the absence of KOH treatment, the obtained carbon sample did not show any 3D structure as shown in Fig. S1(b). This indicates that the chemical treatment with KOH is important to form the surface modified hierarchical HAPC with large surface area. It can be clearly seen that the surfaces of HAPC macropore walls exhibited the meso/nanoporous nature (Fig. 2(c)). Here, the pure HAPC is obtained after acid treatment with HCl as shown in the EDX analysis of Fig. S2. In order to investigate the pore structure of the prepared HAPC, we measured N_2_ adsorption/desorption isotherm as presented in Fig. 2(d). According to IUPAC classifications, the isotherm of the HAPC exhibited a typical type-IV curve, in which an obvious increment in the adsorption at low relative pressures (~0.1 to 0.4) can be found due to the presence of micropores. Similarly, the hysteresis loop observed over the relative pressure range of 0.4–0.9 revealed the presence of mesopores due to the capillary condensation. In addition, a small spike has been observed at the relative pressure close to 1, indicating the presence of macropores^40^. Therefore, the HAPC had a relatively high BET specific surface area of 1589 m^2^ g^−1^. Furthermore, it is worth noting that the size of micropores in the HAPC based on the pore size distribution (inset of Fig. 2(d)) is in agreement with the TEM result (Fig. S1(a)). Such coexistence of porous nature in the prepared HAPC is well correlated with the ideal behavior of supercapacitors because the larger pores provide a basis for fast mass transport of electrolytes whereas the energy storage occurs predominantly in small micropores^41^. Furthermore, the 3D interconnected structure of the HAPC serves as an ideal platform for anchoring other electroactive materials.

**Figure 3:**
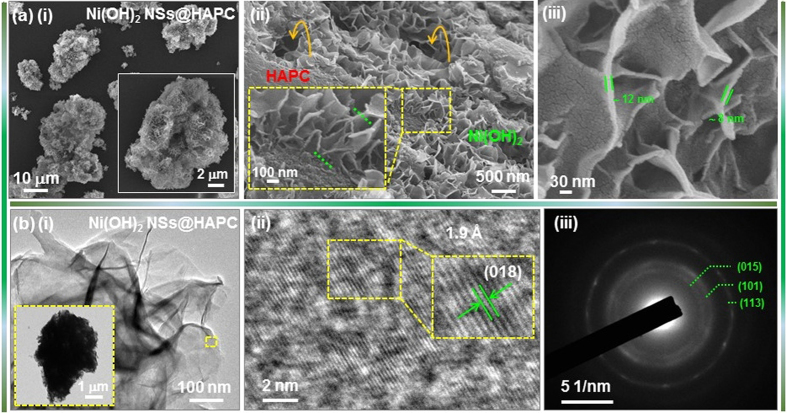
(**a**) (i–iii) FE-SEM images of the Ni(OH)_2_ NSs@HAPC composites synthesized at 90 °C for 10 h. (**b**) (i–iii) TEM images, HR-TEM image and SAED pattern of the corresponding composite materials.

The morphological properties of the Ni(OH)_2_ NSs@HAPC composites are shown in Fig. 3. By mixing the HAPC with nickel salt and hydrolyzing agent, the Ni(OH)_2_ NSs were affluently grafted on the HAPC as shown in Fig. 3(a)(i). The as-obtained hybrid Ni(OH)_2_ NSs@HAPC composites could be expected to provide a high electrical conductivity and penetration paths for electrolyte ions, leading to the enhanced energy storage properties. When compared with the surface of the pristine HAPC, the as-grown Ni(OH)_2_ NSs were completely covered over the whole surface with a vertical alignment and exhibited the height of ∼350–450 nm (Fig. 3(a)(ii)). The arrows shown in Fig. 3(a)(ii) are of the macroporous holes of HAPC. The NSs exhibited ultrathin and porous features with thicknesses of ∼8–12 nm, as shown in Fig. 3(a)(iii). The morphology of the pristine Ni(OH)_2_ NSs under the same solvothermal condition is also shown in Fig. S3. Moreover, the TEM images of the Ni(OH)_2_ NSs@HAPC composites were shown to provide a clear evidence for the conformal coating of Ni(OH)_2_ NSs on HAPC (Fig. 3(b)). From the inset of Fig. 3(b)(i), it can be seen that the Ni(OH)_2_ NSs were compactly adhered to the backbone of HAPC. The NSs were intercalated with each other to form a hierarchical nanonetwork as shown in Fig. 3(b)(i). The high-resolution TEM (HR-TEM) image in Fig. 3(b)(ii) exhibited the lattice fringes with interplanar distance of ∼1.9 Å corresponding to the crystal plane of (018) in Ni(OH)_2_. Also, the selected area electron diffraction (SAED) pattern showed concentric diffraction rings which indicate the polycrystalline nature of the Ni(OH)_2_ (Fig. 3(b)(iii)).

**Figure 4:**
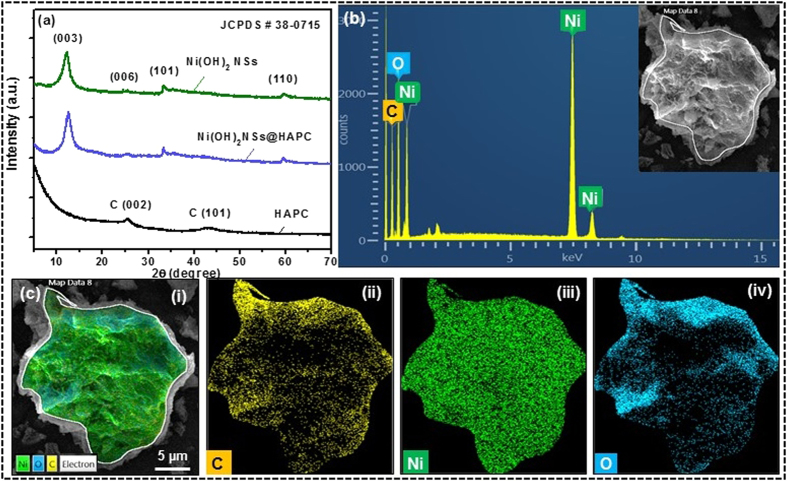
(**a**) XRD patterns of the HAPC and Ni(OH)_2_ NSs@HAPC composites as well as the pristine Ni(OH)_2_ NSs. (**b**) EDX spectrum and (**c**) elemental mapping images of the corresponding composite materials.

The phase composition and crystalline properties of the as-prepared samples were investigated by XRD. As shown in Fig. 4(a), two characteristic peaks observed at 2θ of ∼ 25.6 and 43.7° corresponded to the (002) and (101) reflections of graphitic carbon, respectively. After the growth of Ni(OH)_2_ NSs on HAPC, the main diffraction peaks located at 11.3, 22.7, 33.45 and 59.9° were well indexed to the (003), (006), (101) and (110) crystal planes of the rhombohedral Ni(OH)_2_ (JCPDS card no. 38-0715), respectively. As shown in Fig. S4, the HAPC and Ni(OH)_2_ NSs@HAPC composites were further confirmed by Raman spectroscopy which is an important tool to characterize the carbon-based materials. As can be seen in the spectrum, the broad peaks observed at 1353 and 1590 cm^−1^ are related to the D-band and G-band, respectively. The presence of these bands is due to the disordered carbon structure and the graphitic or aromatic structures (sp^2^ hybridized) of the HAPC. Additionally, the Ni(OH)_2_ peaks were observed at 460, 711 and 1045 cm^−1^, indicating that the Ni(OH)_2_ NSs were adhered to the HAPC microparticles. Furthermore, the EDX spectrum of the Ni(OH)_2_ NSs@HAPC revealed the constituent Ni and O elements along with C, as observed from Fig. 4(b). From the elemental mapping images, the regions of the Ni and O elements were uniformly overlapped on the HAPC skeleton, confirming that the Ni(OH)_2_ NSs were well distributed on the surface of HAPC (Fig. 4(c)). Such synergistic effect of combining highly porous and conductive carbon with electrically conductive pseudocapacitive Ni(OH)_2_ may generate higher energy storage properties than the single components or phases. The surface area of the Ni(OH)_2_ NSs@HAPC composite was analyzed by Brunauer–Emmett–Teller (BET) N_2_ adsorption–desorption isotherms and the obtained results are shown in Fig. S3. Compared to the HAPC (1589 m^2^g^−1^), the Ni(OH)_2_ NSs@HAPC nanocomposite exhibited a lower surface area (80.3 m^2^g^−1^), as expected due to the decoration of Ni(OH)_2_ NSs (Fig. S5).

**Figure 5:**
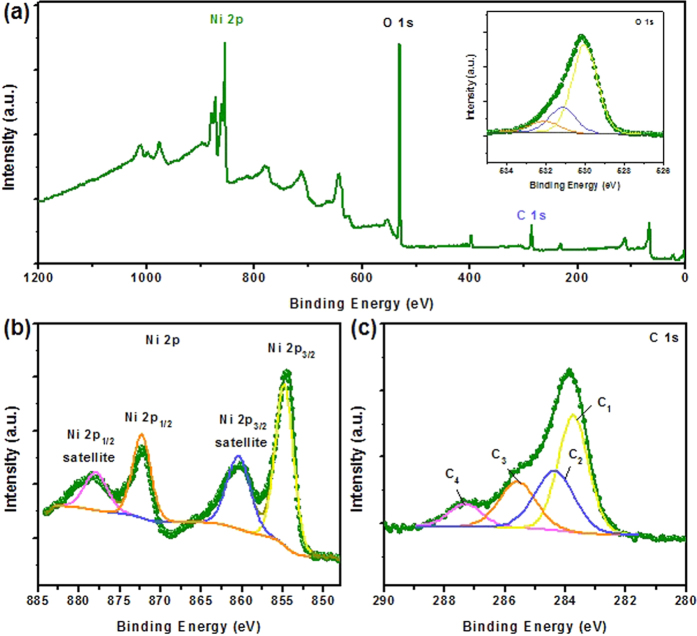
(**a**–**c**) XPS results of the Ni(OH)_2_ NSs@HAPC composite materials.

A more detailed analysis on the surface elemental composition and chemical state of the Ni(OH)_2_ NSs@HAPC composites was performed by XPS and the corresponding results are shown in Fig. 5(a–c). The total-survey scan XPS spectrum of the Ni(OH)_2_ NSs@HAPC confirmed the existence of C, Ni and O (Fig. 5(a)). From the Fig. 5(b), the high-resolution core-level spectra of Ni 2p could be fitted with two spin-orbit doublets and two shakeup satellites using a Gaussian fitting method. The peaks located at around 854.8 and 872 eV are related to the Ni 2p_3/2_ and Ni 2p_1/2_ spin-orbit doublets, indicating that the Ni species are in the +2 oxidation state^42^. The peaks at around 860.5 and 878.1 eV are attributed to the shakeup satellite peaks of Ni 2p_3/2_ and Ni 2p_1/2_ in Ni 2p. In the high-resolution XPS spectra of the C 1 s region (Fig. 5(c)), the peaks at around 283.8 (C_1_), 284.4(C_2_), 285.6(C_3_) and 287.4 eV (C_4_) were observed, corresponding to the C-H, C-C, C-OH and C = O (carbonyl/quinone groups), respectively. Similarly, the binding energy peaks of the O 1 s region (528–532 eV) correspond to the carbonyl/quinone, carbonate structure and hydroxyl/ethers (C-OH/C-O-C), or Ni-OH, respectively (inset of Fig. 5(b)). These results further verify that the main elements of C, Ni and O are presented in Ni(OH)_2_ NSs@HAPC composites.

**Figure 6:**
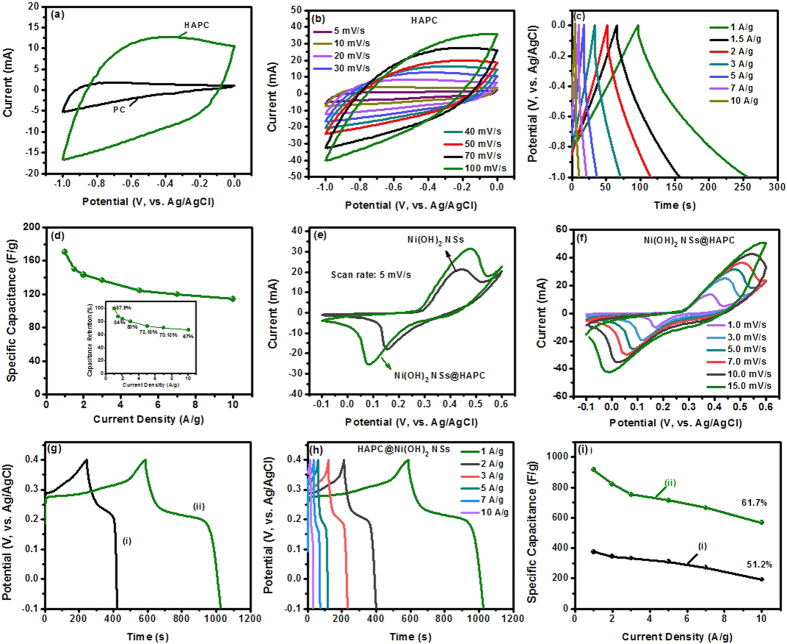
Electrochemical performance of the synthesized HAPC, Ni(OH)_2_ NSs@HAPC composites and the pristine Ni(OH)_2_ NSs in 1 M of KOH electrolyte solution. (**a**) Comparative CV curves of the HAPC and PC at a scan rate of 30 mV/s. (**b**–**d**) CV, GCD curves and specific capacitance values of HAPC. (**e**) CV curves of the Ni(OH)_2_ NSs@HAPC composites and the pristine Ni(OH)_2_ NSs recorded at a constant scan rate of 5 mV/s. (**f**) CV curves of the Ni(OH)_2_ NSs@HAPC composites at various scan rates. (**g**) GCD curves of the (i) Ni(OH)_2_ NSs and (ii) Ni(OH)_2_ NSs@HAPC composites at a current density of 1 A/g. (**h**) GCD curves Ni(OH)_2_ NSs@HAPC composites electrode at various current densities. (**i**) Calculated specific capacitance values for the (i) pristine Ni(OH)_2_ NSs and (ii) Ni(OH)_2_ NSs@HAPC composites at various current densities.

With the advantage of internal mesoporous structure in HAPC and hierarchically-grown Ni(OH)_2_ NSs on the HAPC, the composites could be highly endorsed to provide remarkable energy storage properties for use as electroactive materials in supercapacitors. To test the feasibility, the electrochemical performances of the PC, HAPC, Ni(OH)_2_ NSs@HAPC and Ni(OH)_2_ NSs, individually, were investigated for potential applications as supercapacitor electrodes. The electrochemical properties (Fig. 6) of the electroactive materials coated CC were evaluated in a three-electrode system in 1 M of KOH solution. Figure 6(a) shows the CV curves of the PC and HAPC electrodes at a scan rate of 30 mV/s in the potential range of −1 to 0 V. As shown in Fig. 6(a), the CV curve of the HAPC electrode exhibited a typical rectangular shape and the integral area under the curve was relatively higher than that of the PC electrode (i.e., without chemically treated pine carbon), indicating an excellent electrochemical performance. This is mainly ascribed to the large surface area and porous nature of the HAPC, which allows for the accessibility of the electrolyte ions into its interior parts. The CV curves of the HAPC were tested at various scan rates of 5 to 100 mV/s as shown in Fig. 6(b). Even at higher scan rates, the CV curves retained quasi-rectangular shapes, indicating the ideal capacitive behavior and good conductivity of the HAPC. The performance of supercapacitor electrodes in real applications was predominantly estimated by their GCD curves under constant currents, which reveals their energy storage properties. Figure 6(c) shows the GCD curves of the HAPC sample at various current densities of 1 to 10 A/g in 1 M of KOH solution. All the GCD curves exhibited isosceles triangle shapes, further suggesting the EDLCs behavior of the carbon-based materials. According to the discharge times in Fig. 6(c), the specific capacitance values were calculated and the obtained results as a function of applied current density are plotted in Fig. 6(d). At different current densities of 1, 1.5, 2, 3, 5, 7 and 10 A/g, the HAPC exhibited the specific capacitance values of 170.4, 149.8, 143.2, 136.5, 124.7, 119.5 and 114.2 F/g, respectively. It is noticeable that the HAPC showed a good capacitance retention of 67% at a current density of 10 A/g (inset of Fig. 6(d)), indicating the good rate capability of the material.

Furthermore, the electrochemical performance of the Ni(OH)_2_ NSs@HAPC composites was also evaluated and compared to that of the Ni(OH)_2_ NSs prepared without mixing of HAPC during the solvothermal process. Figure 6(e) displays the CV curves of the Ni(OH)_2_ NSs@HAPC and pristine Ni(OH)_2_ NSs at a scan rate of 5 mV/s in 1 M of KOH electrolyte solution. Both the electrodes showed a pair of redox peaks, evidently demonstrating that the capacitance characteristics are mainly governed by pseudocapacitive behavior (Faradaic reactions) of the electrode materials. By depositing Ni(OH)_2_ NSs on the surface of HAPC conductive scaffolds, the Ni(OH)_2_ NSs@HAPC composites exhibited the relatively larger peak current values than that of the pristine Ni(OH)_2_ NSs, resulting in the better pseudocapacitive behavior. Thus, much higher specific capacitance could be expected for the composite materials. The redox peaks in CV curves during the electrochemical process could be explained by the following reversible reactions:^30^,^43^


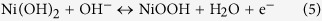


Figure 6(f) shows the CV curves of the Ni(OH)_2_ NSs@HAPC composites at various scan rates of 1 to 15 mV/s. With increasing the scan rate from 1 to 15 mV/s, the shape of the CV curves remains almost the same, but the oxidation peak shifted to the higher potential, whereas a reduction peak moved to the lower potential. Such phenomenon of the Ni(OH)_2_ NSs@HAPC composites during the increased scan rate signifies that the kinetics of the interfacial faradaic redox reactions and the rate of electronic and ionic transports are rapid enough^44^. To estimate the specific capacitance values, the GCD measurements were further carried out for the Ni(OH)_2_ NSs@HAPC composites and pristine Ni(OH)_2_ NSs electrodes, as shown in Fig. 6(g) and (h). Both the curves including two separated plateaus in the charge-discharge process, which are mainly related to the reversible redox reactions of the electrode materials. This pseudocapacitive behavior in GCD curves is in good agreement with the CV curves. However, the charge-discharge time of the Ni(OH)_2_ NSs@HAPC composite electrode was higher compared to the pristine Ni(OH)_2_ NSs. Such higher GCD plateau in Ni(OH)_2_ NSs@HAPC composite is due to the excellent electrical conductivity of the electroactive materials, which provide the convenient channels for the penetration of electrolyte ions and transport of electrons. Herein, the mass of the electroactive materials on CC was measured to be around 2.0 ± 0.05 mg. On the basis of GCD curves at different current densities, the calculated specific capacitance (C_sp_) values for the both materials as a function of applied current density are plotted in Fig. 6(i). At a current density of 1 A/g, the as-prepared Ni(OH)_2_ NSs@HAPC composites showed a maximum specific capacitance of 916.4 F/g, whereas the pristine Ni(OH)_2_ NSs had a specific capacitance of 375.1 F/g. The C_sp_ values of the composite materials were 916.4, 820.55, 753.64, 713.94, 665.63 and 565.71 F/g at different current densities of 1, 2, 3, 5, 7 and 10 A/g, respectively, with a capacitance retention of 61.7%. It is clear that the HAPC as conductive scaffolds plays a promising role in the composite materials to enhance the energy storage properties. The obtained specific capacitance values in this work are comparable and even higher than the previously reported electroactive materials for supercapacitors (Table S1).

**Figure 7:**
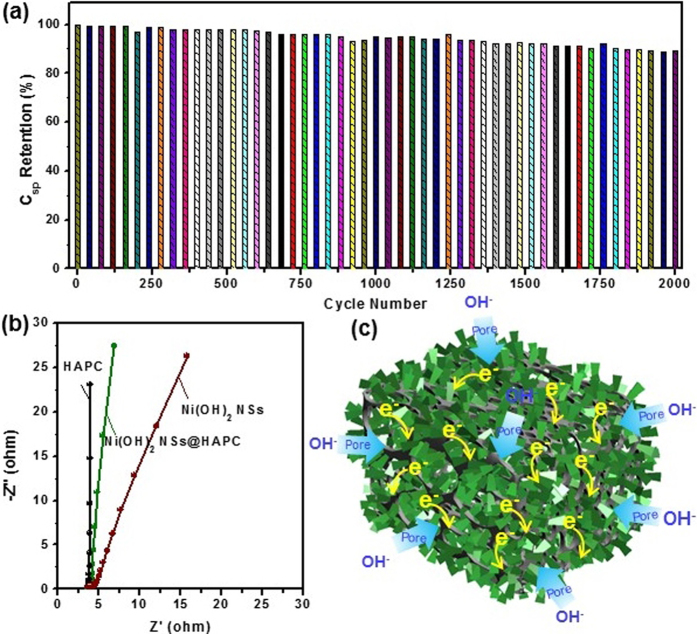
(**a**) Long-term cycling process of the Ni(OH)_2_ NSs@HAPC composites at a current density of 5 A/g, (**b**) EIS curves of the HAPC, Ni(OH)_2_ NSs@HAPC and pristine Ni(OH)_2_ NSs in 1 M of KOH electrolyte solution in the frequency range of 0.01 to 100 kHz. (**c**) Schematic illustration showing the merits of Ni(OH)_2_ NSs@HAPC composites during the electrochemical measurement.

Ultimately, long-life cyclic stability of the electrode is another important factor in relation to the energy storage performance of the supercapacitors. Accordingly, the cycling process was investigated at a current density of 5 A/g in 1 M of KOH solution in the potential range of −0.1 to 0.4 V. The measured specific capacitance values as a function of cycle number are shown in Fig. 7(a). After 2000 cycles, the capacitance retention of the composite electrode was about 89.5%. The first GCD curves of the Ni(OH)_2_ NSs@HAPC composites (Fig. S5) revealed the stable charge-discharge times, indicating the reliable electrochemical reversibility for the composite electrode. Such excellent cyclic stability of the composite materials could be mainly ascribed to the hierarchical structure and porous property. In addition, the EIS analysis was also performed to investigate the ion and electron transport kinetics of the electroactive materials. Herein, the Nyquist plots of the samples were carried out in the frequency range of 0.01 Hz to 100 kHz at an amplitude of 10 mV using 1 M KOH solution. As shown in Fig. 7(b), the Nyquist plots of the HAPC, Ni(OH)_2_ NSs@HAPC and Ni(OH)_2_ NSs exhibited one semicircle in the high frequency region and a linearly sloped line in the low frequency region, respectively. From the EIS curves, the charge transfer resistances (R_ct_) of the HAPC, Ni(OH)_2_ NSs@HAPC and Ni(OH)_2_ NSs were estimated to be about 3.7, 3.86 and 4.31 Ω, respectively. Compared to the Ni(OH)_2_ NSs@HAPC and Ni(OH)_2_ NSs, the HAPC appears to be slightly smaller semicircle because of its lower electrical conductivity. Thus, the EIS results once again reveal that the HAPC is the excellent scaffolds, which improves the electrochemical performance when the electroactive materials are grown on it. The schematic diagram in Fig. 7(c) illustrates the synergistic effect of the highly porous and hierarchical Ni(OH)_2_ NSs pillared HAPC composites for high-performance supercapacitor electrodes. Here, the HAPC acts as a conductive scaffold for fast electron transport. Moreover, the Ni(OH)_2_ NSs@HAPC composites provide the free paths for effective diffusion of electrolyte ions, which enhances the surface wettability and improves the electrochemical activity. Once the electrolyte ions easily diffuse into the composite material, sufficient faradaic redox reactions occur by the generation of electrons on current collector through the conductive scaffolds. As a result, the enhanced electrochemical properties were obtained with a high rate capability. Consequently, it is suggested that the Ni(OH)_2_ NSs@HAPC composites could be expected to offer a potential use as electroactive materials for various energy storage devices.

## Conclusion

In summary, novel and cost-effective Ni(OH)_2_ NSs@HAPC composites were successfully synthesized by the utilization of biomass source as a conducting skeleton. By means of alkali treatment and carbonization processes, the HAPC powder with meso-, micro- and nano-porous nature was facilely prepared using pine cone powder as a biomass source. Meanwhile, the conducting skeleton of HAPC powder was then mixed with nickel salt and hydrolyzing agent, thereby leading to the formation of hierarchical ultrathin Ni(OH)_2_ NSs@HAPC composites under the solvothermal condition. When applied as electroactive materials for supercapacitors, the composite materials exhibited a relatively higher specific capacitance value of 916.4 F/g at 1 A/g compared to the pristine HAPC and Ni(OH)_2_ NSs. The enhanced energy storage properties of the Ni(OH)_2_ NSs@HAPC could be mainly attributed to the large surface area of the HAPC and sufficient faradaic redox reactions of the Ni(OH)_2_ NSs. This facile process for the utilization of biomass source to produce the cost-effective carbon-based materials and their composites with transition metal oxides is expected to be very promising in energy-related devices.

## Additional Information

**How to cite this article:** Nagaraju, G. *et al*. Ultrathin nickel hydroxide nanosheet arrays grafted biomass-derived honeycomb-like porous carbon with improved electrochemical performance as a supercapacitive material. *Sci. Rep.* **7**, 45201; doi: 10.1038/srep45201 (2017).

**Publisher's note:** Springer Nature remains neutral with regard to jurisdictional claims in published maps and institutional affiliations.

## Supplementary Material

Supporting Information
