# Supplementary material for: Ultrathin nickel hydroxide nanosheet arrays grafted biomass-derived honeycomb-like porous carbon with improved electrochemical performance as a supercapacitive material
Source: Sci Rep. 2017 Mar 24;7:45201. doi: 10.1038/srep45201 (PMC5364545; doi:10.1038/srep45201)
Supplement: Supporting Information [file srep45201-s1.pdf]

Supporting Information

Ultrathin nickel hydroxide nanosheet arrays grafted biomass-derived honeycomb-like porous carbon with improved electrochemical performance as a supercapacitive material

Goli Nagaraju, Sung Min Cha and Jae Su Yu*

Department of Electronic Engineering, Institute for Wearable Convergence Electronics, Kyung Hee University, 1 Seocheon-dong, Giheung-gu, Yongin-si, Gyeonggi-do 446-701, Republic of Korea

*Address correspondence to jsyu@khu.ac.kr

Tel: +82-31-201-3820; Fax: +82-31-206-2820

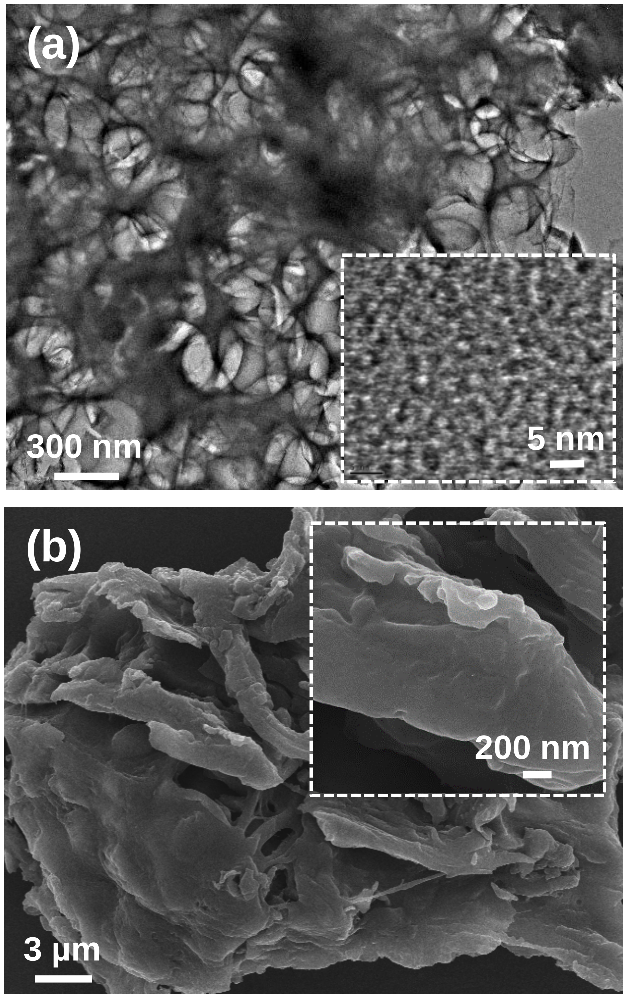

Figure S1. (a) TEM images of the HAPC and FE-SEM image of the PC prepared without activating with KOH.

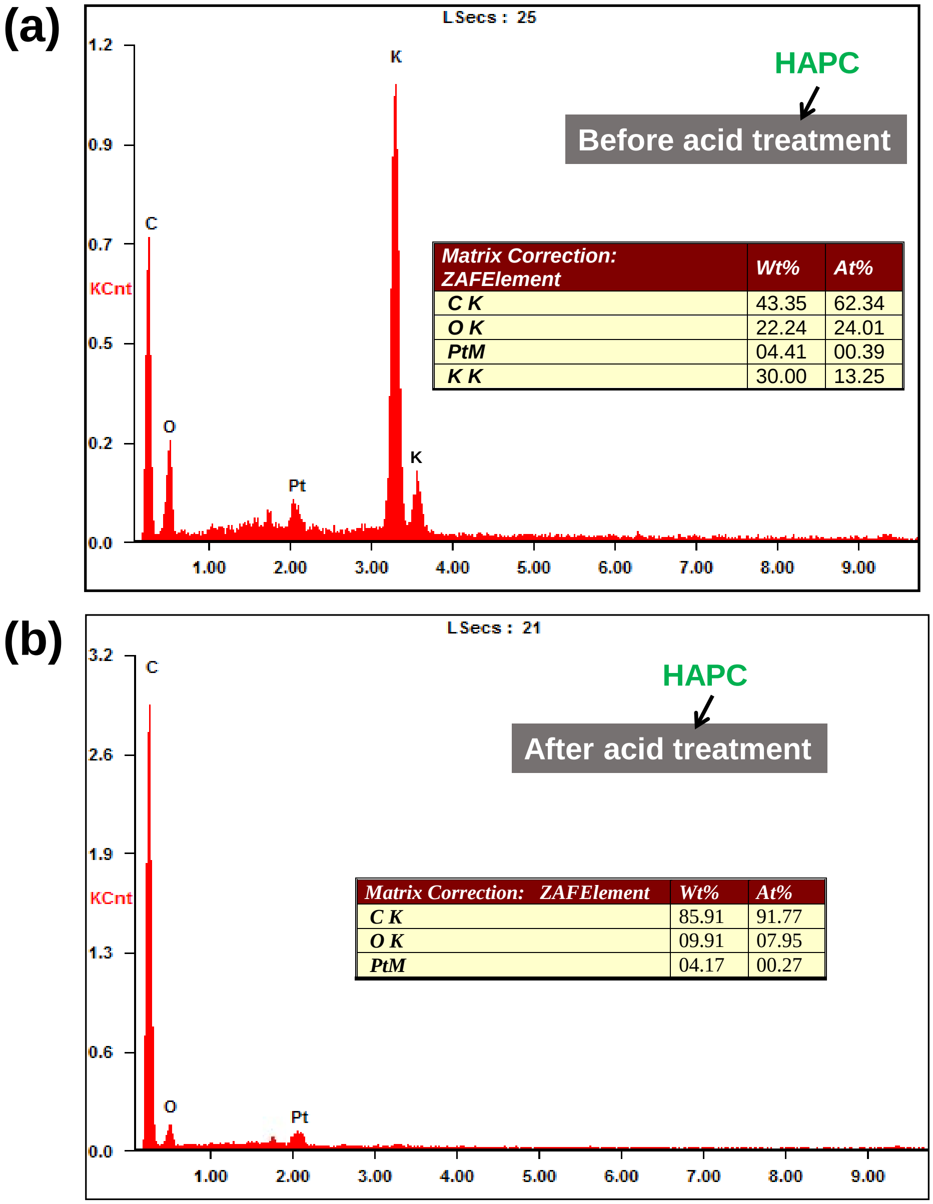

Matrix Correction: ZAFElement	Wt%	At%
C K	43.35	62.34
O K	22.24	24.01
PtM	04.41	00.39
K K	30.00	13.25

Matrix Correction: ZAFElement	Wt%	At%
C K	85.91	91.77
O K	09.91	07.95
PtM	04.17	00.27

Figure S2. EDX analysis of the HAPC before and after acid treatment.

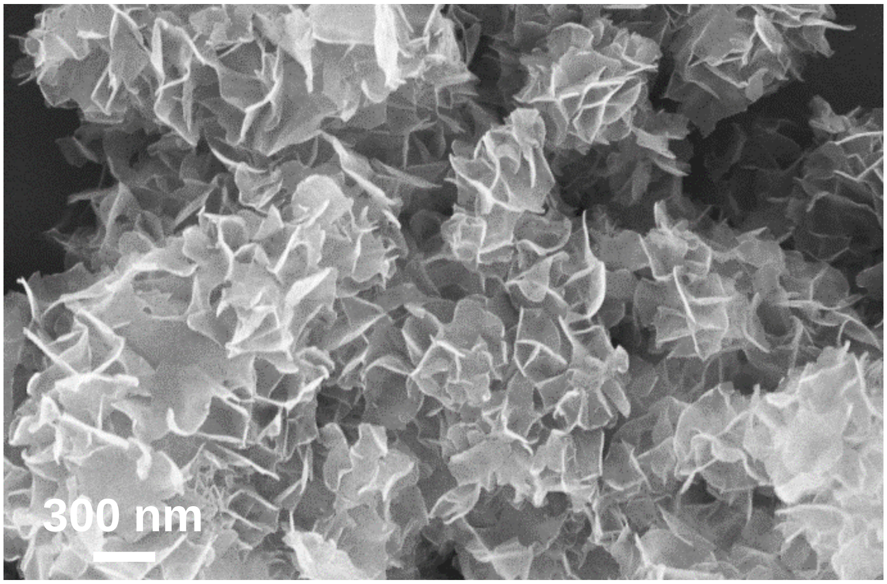

Figure S3. FE-SEM image of the prepared $Ni(OH)_2$ NSs without adding HAPC powder.

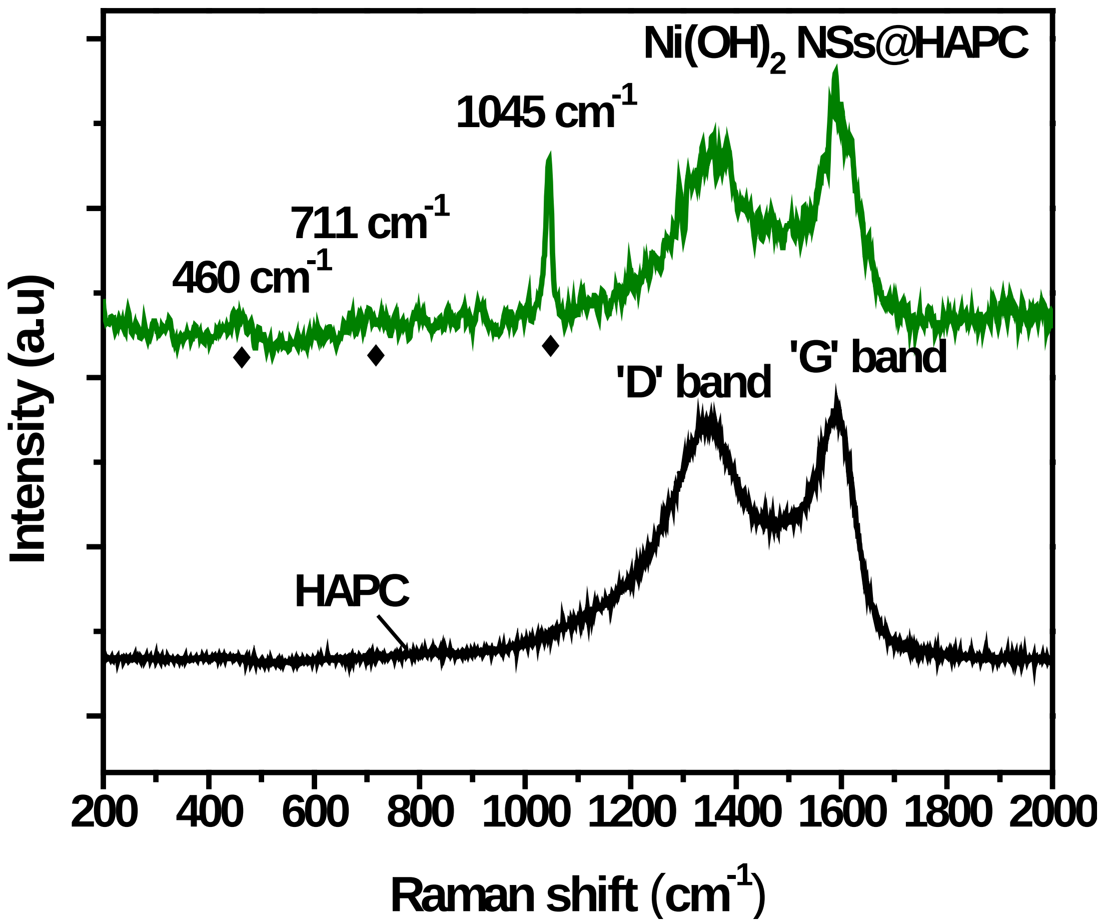

Figure S4. Raman spectra of the HAPC and $Ni(OH)_2$@HAPC composite materials.

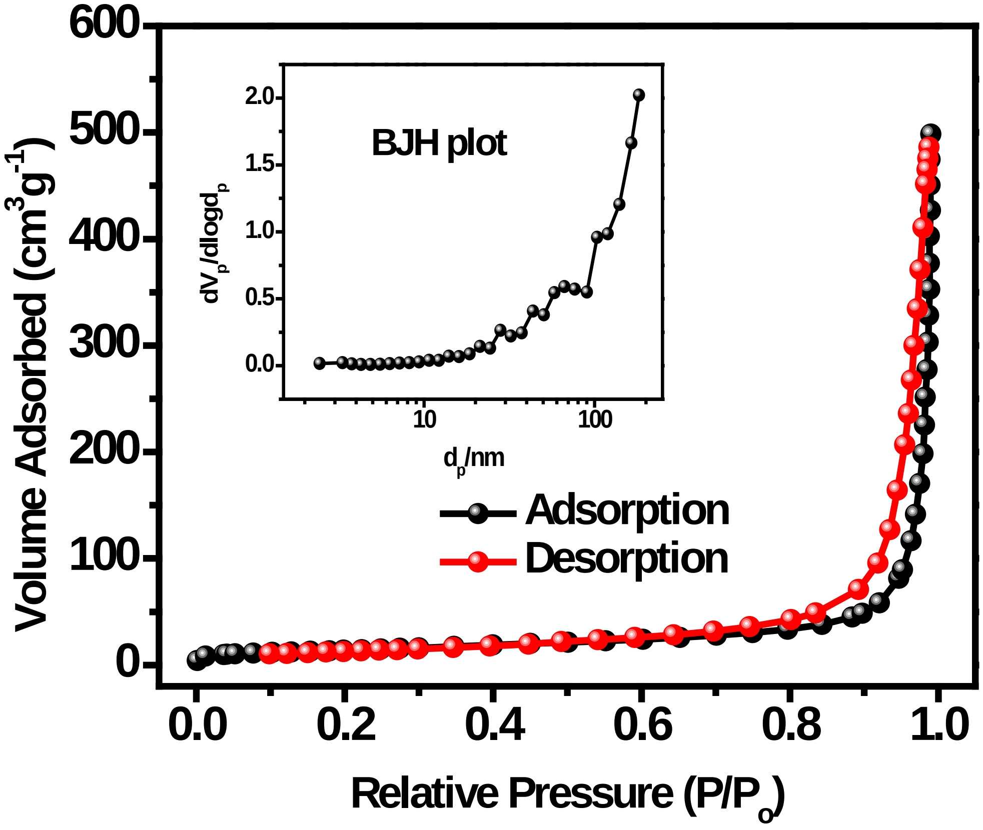

Figure S5. BET surface area of the $Ni(OH)_2$@HAPC composite.

Table S1: Electrochemical performance of the previously reported materials with $Ni(OH)_2$@HAPC composite.

**Electrode material**	**Current collector**	**Synthesis method**	**Electrolyte**	**Test condition**	**Specific capacitance**	**Ref.**
NiO nanodots	Ni foam	High-temperature solution phase	6 M KOH	2 A/g	856 F/g	S1
$Ni(OH)_2$ nanostructures	Carbon paper	Reflux process	2 M KOH	1 A/g	332 F/g	S2
NiO nanotubes	Ni foam	Electro-deposition	2 M KOH	2 A/g	675 F/g	S3
Porous NiO film	Ni foil	CBD method	1 M KOH	1 A/g	309 F/g	S4
MnO_2@bio carbon	Ni foam	Hydrothermal	1 M Na_2SO_4	0.2 A/g	262.2 F/g	S5
$Ni(OH)_2$@car-bon nanotubes	Ni grid	CBD method	6 M KOH	0.4 A/g	190 mAh/g	S6
$Ni(OH)_2$@activated carbon	Ni foam	-------	6 M KOH	1 A/g	530 F/g	S7
rGO@Ni nanoparticles	Carbon cloth	Hydrothermal	0.05 M PBS+KCl+ NaH_2PO_4	0.2 A/g	70 F/g	S8
NiO hollow spheres	Ni foam	CBD method	2 M KOH	1 A/g	346 F/g	S9
NiO film	Ni foam	Ammonia evaporation method	1 M KOH	2 A/g	232 F/g	S10
Nickel oxide @hydroxide	Stainless steel	Chemical precipitation method	0.5 M KOH	10 mV/s	108 F/g	S11
$Ni(OH)_2$@HAPC composite	Carbon cloth	Solvothermal method	1 M KOH	1 A/g	916.4 F/g	This work

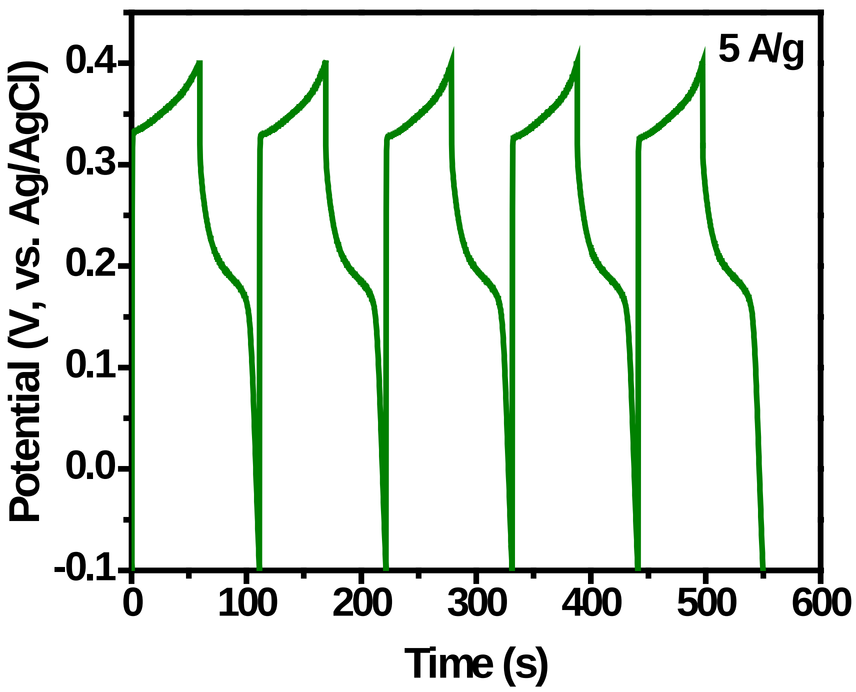

Figure S6. First five GCD cycles of the $Ni(OH)_2$@HAPC composite measured at a current density of 5 A/g.

References

1 Zheng, X. et al. 3D interconnected macro-mesoporous electrode with self-assembled NiO nanodots for high-performance supercapacitor-like Li-ion battery. Nano Energy 22, 269-277, doi:http://dx.doi.org/10.1016/j.nanoen.2016.02.017 (2016).

2 Weihong, L., Jieyi, Y., Tayirjan, T. I., De-Quan, Y. & Edward, S. Aqueous synthesis and growth of morphologically controllable, hierarchical Ni(OH) 2 nanostructures. Materials Research Express 2, 075011 (2015).

3 Cao, F., Pan, G. X., Xia, X. H., Tang, P. S. & Chen, H. F. Synthesis of hierarchical porous NiO nanotube arrays for supercapacitor application. J. Power Sources 264, 161-167, doi:http://dx.doi.org/10.1016/j.jpowsour.2014.04.103 (2014).

4 Xia, X.-h., Tu, J.-p., Wang, X.-l., Gu, C.-d. & Zhao, X.-b. Hierarchically porous NiO film grown by chemical bath deposition via a colloidal crystal template as an electrochemical pseudocapacitor material. J. Mater. Chem. 21, 671-679, doi:10.1039/c0jm02784g (2011).

5 Li, Y. et al. Fabrication of manganese dioxide nanoplates anchoring on biomass-derived cross-linked carbon nanosheets for high-performance asymmetric supercapacitors. J. Power Sources 300, 309-317, doi:http://dx.doi.org/10.1016/j.jpowsour.2015.09.077 (2015).

6 Wang, Y.-g., Yu, L. & Xia, Y.-y. Electrochemical Capacitance Performance of Hybrid Supercapacitors Based on $Ni(OH)_2$ ⁄ Carbon Nanotube Composites and Activated Carbon. J. Electrochem. Soc. 153, A743-A748, doi:10.1149/1.2171833 (2006).

7 Park, J. H., Park, O. O., Shin, K. H., Jin, C. S. & Kim, J. H. An Electrochemical Capacitor Based on a Ni (OH) 2/Activated Carbon Composite Electrode. Electrochemical and Solid-State Letters 5, H7-H10, doi:10.1149/1.1432245 (2002).

8 Valipour, A., Hamnabard, N. & Ahn, Y.-H. Performance evaluation of highly conductive graphene (RGOHI-AcOH) and graphene/metal nanoparticle composites (RGO/Ni) coated on carbon cloth for supercapacitor applications. RSC Adv. 5, 92970-92979, doi:10.1039/c5ra14806e (2015).

9 Yan, X. et al. Rational synthesis of hierarchically porous NiO hollow spheres and their supercapacitor application. Mater. Lett. 95, 1-4, doi:http://dx.doi.org/10.1016/j.matlet.2012.12.073 (2013).

10 Zhang, Y. Q. et al. Self-assembled synthesis of hierarchically porous NiO film and its application for electrochemical capacitors. J. Power Sources 199, 413-417, doi:http://dx.doi.org/10.1016/j.jpowsour.2011.10.065 (2012).

11 Wu, M.-S. & Hsieh, H.-H. Nickel oxide/hydroxide nanoplatelets synthesized by chemical precipitation for electrochemical capacitors. Electrochim. Acta 53, 3427-3435, doi:http://dx.doi.org/10.1016/j.electacta.2007.12.005 (2008).
